# Feed supplementation with potentiated zinc and/or tannin-rich extracts reduces ETEC infection severity and antimicrobial resistance genes in pig

**DOI:** 10.3389/fvets.2025.1494103

**Published:** 2025-02-21

**Authors:** Catherine Ollagnier, Maria-Rita Mellino, Nicolas Pradervand, Marco Tretola, Sebastien Dubois, Stephane Durosoy, Olivier Desrues, Johana Bellon

**Affiliations:** ^1^Pig Research Unit, Agroscope, Posieux, Switzerland; ^2^Department of Agricultural Sciences, University of Sassari, Sassari, Italy; ^3^Feed Biology, Agroscope, Posieux, Switzerland; ^4^Animal Biology, Agroscope, Posieux, Switzerland; ^5^Feed Chemistry, Agroscope, Posieux, Switzerland; ^6^Animine Precision Minerals, Annecy, France; ^7^Silvateam, San Michele Mondovì, Italy

**Keywords:** *Escherichia coli*, post-weaning diarrhea, resistome, tannins, zinc oxide

## Abstract

Most antimicrobials used in pig production are prescribed to treat post-weaning diarrhea (PWD), which constitutes a major health issue in pig production. With the spread of multidrug-resistant pathogens, finding solutions to diminish the severity of PWD without antibiotics has become increasingly critical. Potentiated forms of zinc oxide (ZnO) and plant-based bioactive compounds like tannins have been shown to alleviate the severity of diarrhea, thus reducing the need for antibiotic treatment. The aim of this project was to test whether a potentiated form of ZnO (pZnO), alone (study 1) or in combination with tannin-rich extract (study 2), can be used in starter diets for weaned piglets infected by enterotoxigenic *Escherichia coli* (ETEC) to reduce PWD severity. At 26 ± 1.6 days of age (average body weight 7.8 ± 1.02 kg), 160 piglets (study 1, *n* = 72; study 2, *n* = 88) were randomly and equally assigned to four dietary treatments (study 1 = 18; study 2 = 22 pigs/group) and orally infected 4 days after weaning with a solution containing 10^10^ ETEC F4. Study 1 compared the effect of 150 mg Zn/kg pZnO (pZnO-150) and 300 mg Zn/kg pZnO (pZnO-300) to a negative control (C) and a positive control, 3,000 mg Zn/kg ZnO (C-3000). In study 2, a combination of 7.5 g/kg tannin extract (quebracho and chestnut) and 150 mg Zn/kg pZnO (TAN+pZnO-150) was compared to pZnO-150 and 7.5 g/kg tannin-rich extract (TAN) alone and to a negative control (C). The fecal score, bodyweight, daily food intake per pen, fecal F4 ETEC and Zn levels were analyzed. The small intestine content was sampled 9 days after infection to analyze the number of antimicrobial resistance genes. Regardless of the inclusion level, TAN+pZnO-150, TAN and pZnO led to a reduction in antibiotic treatment (*p* < 0.05), but only TAN and TAN+pZnO-150 reduced the fecal score (*p* < 0.05). C-3000 improved the average daily gain (*p* < 0.05). Tannin-rich extract and potentiated zinc oxide (pZnO) in starter diets effectively reduce the need for antibiotics in ETEC-challenged piglets. Traditional high-dose ZnO improved growth rates, but lower-dose alternatives with tannins provided health benefits without high zinc levels. These findings highlight sustainable dietary strategies to manage post-weaning diarrhea, supporting reduced antibiotic use in pig production.

## 1 Introduction

The way antibiotics (AB) are used in animal production has changed in the last 20 years due to the emerging threat of bacteria that are resistant to antibiotics. European Union first banned AB as feed additives for growth promotion in 2003 (1) and then prohibited their use as a prophylactic treatment for groups of animals in 2018 (2). Throughout Europe, governments are asking for prudent use of AB, and this approach has been fruitful. In Denmark, the AB use in animals decreased by 14% between 2013 and 2018 (3). Many European countries continue to establish action plans to further reduce, refine and replace the use of AB. In this context, management and breeding practices are changing, with alternatives to AB being explored.

In pig production, the majority of AB are used during the post-weaning period. In a study on 227 farms in Switzerland (4), 421 kg of active AB ingredients were used for pigs, 49.4% of which were used for weaners. The post-weaning period is critical for pigs. During this transition period, they are separated from their mothers and regrouped in a new environment, with a new drinking and feeding system. Their digestive tract has to adapt to a new diet composition. All these changes generate stress and lead to an intestinal dysbiosis that can cause diarrhea (5). Post-weaning diarrhea (PWD) is defined as a diarrhea occurring within 14 days after weaning (6). This digestive disorder does not always have an infectious component, but it may be complicated by enterotoxigenic *Escherichia coli* (ETEC) infection (7). ETEC are pathogenic *E. coli* that adhere to the small intestine mucosae, with F4 and F18 being the two main types of fimbriae. After adhesion to the mucosa, ETEC F4 secrete two types of toxins: heat stable (STa and STb) and labile toxins (LT).

High doses of zinc oxide (ZnO) were found to be an effective solution to replace AB use for digestive disorders in pigs. ZnO doses higher than 2,500 mg Zn/kg feed reduce diarrhea and improve feed intake, bodyweight (BW) gain and feed conversion rate (8). However, due to the non-volatile and non-degradable physicochemical properties of Zn, a continuous field application of manure from animals treated with a high dose of ZnO leads to a gradual and continuous accumulation of Zn in soil, water and sediments. A field study in Denmark reported an increase of >45% in Zn concentrations in soil from 1998 to 2014 (9). Zn might also promote the spread of antimicrobial resistance (10–12). Indeed, the level of Zn in liquid pig manure correlates with the level of antimicrobial resistance (13). Ultimately, the European Union decided in 2017 to ban the use of high doses of ZnO as veterinary medicinal product (14) because the benefits of ZnO in terms of preventing diarrhea in pigs did not outweigh the risks for the environment. Anticipating that the European ban on high doses of ZnO will become effective in 2022, new formulations of ZnO have been explored to provide a therapeutic effect at doses accepted by the regulatory authorities. Previous studies have demonstrated that a potentiated form of ZnO (pZnO) could improve zootechnical performance and reduce the severity of diarrhea when given to weaning pigs (15–18).

Plant polyphenolic compounds, such as those in plant extracts, have great value as a natural source of bioactive compounds to alleviate digestive disorders in pigs (19). In the polyphenol group, tannins are the most important class and are mainly classified into two subgroups: hydrolysable and condensed tannins (20). Tannins extracted from chestnut trees (*Castanea sativa* Mill.) and quebracho trees (*Schinopsis* spp.) contain hydrolysable and condensed tannins, respectively. Those tannin extracts have been tested in pigs for their antioxidant, anti-inflammatory and antibacterial activities, both on conventional and ETEC-infected weaners (21–26).

This study evaluated the efficacy of a potentiated form of ZnO with or without a chestnut and quebracho tannin-rich extract in reducing the severity of PWD in an enterotoxigenic *Escherichia coli* infection model. The effects of these dietary treatments on health status, growth performance, excretion of pathogens, zinc and antimicrobial resistance genes were assessed.

## 2 Materials and methods

### 2.1 Animals, experimental design and dietary treatment

Two studies were carried out to test the efficacy of two ZnO sources supplemented at different inclusion levels with or without tannin extracts in reducing PWD after an ETEC F4 challenge. In study 1, four starter diets were prepared: the negative control (C) and positive control (C-3000) diets were supplemented with 150 and 3,000 mg/kg ZnO (zinc oxide, Millenis SAS, France), respectively, whereas the pZnO-150 and pZnO-300 diets were supplemented with 150 or 300 mg/kg pZnO (HiZox^®^, Animine, Annecy, France), respectively. The HiZox^®^ formulation contained 94% ZnO. In study 2, we used two diets in addition to the C and pZnO-150 diets: the TAN diet was supplemented with 7.5 g/kg of a tannin extract containing chestnut and quebracho tannins (Silvafeed^®^Nutri P, Silvateam, Italy), and the TAN+pZnO-150 diet was supplemented with 7.5 g/kg of the tannin extract plus 150 mg/kg of pZnO. The Silvafeed^®^Nutri P product contained a minimum of 75% tannins, as measured by the ISO 14088 standard. The standard starter diet was formulated in accordance with Swiss feeding guidelines (27) for weaned pigs (Supplementary Table S1). The doses of pZnO and tannins riche extract were selected based on literature (18, 19). Both experiments were approved by the Swiss cantonal veterinary office under the application numbers 29,361 and 31,400, respectively. The model of infection with ETEC F4 had been previously validated (22).

Female and castrated male Swiss Large White piglets, originating from the Agroscope sows' herd, were weaned at 26 (±1.6) days (mean ± standard deviation) with an average bodyweight of 7.8 ± 1.02 kg. A total of 72 piglets participated in study 1, while study 2 included 88 piglets, resulting in 18 and 22 piglets per group, respectively. All piglets were determined to be sensitive to ETEC F4, using a previously described method (28). Briefly, an ear punch (3 mm in diameter) was collected from 420 piglets before weaning. After DNA extraction, PCR was carried out using primers coding for flanking markers of F4acR localized near the MUC13 gene. Piglets having at least one copy of the genetic marker for ETEC F4 receptor were considered sensitive to ETEC F4 infection.

For each study, piglets within each litter were randomly allocated to one of four dietary treatments based on their body weight. Animals were housed in pens (2.6 m^2^) in groups of three to four animals, all belonging to the same dietary treatment group. Fresh feed was offered once a day, and leftovers were weighed daily. Water was available *ad libitum*, and an electrolyte solution was provided to limit the risk of dehydration.

Four days after weaning, all piglets were infected (d0) with an oral solution containing 10^10^ CFU ETEC F4. Trained staff monitored the piglets' health at least once a day. Nine (±1) days after infection, all piglets, except 4 piglets per group in study 2, were euthanized by electronarcosis followed by exsanguination, and samples of intestinal content and jejunum mucosa were collected.

A total of three farrowing series were necessary to achieve the requested number of piglets for each study. There were two sudden deaths in study 1, one in group C and one in C-3000. The reasons for these deaths are unknown, as these pigs did not have high fecal scores the day before. Two pigs (one in group C and one in group pZnO-150, study 1) had to be removed due to arthritis and panaris, respectively. One pZnO-150 pig in study 2 had to be euthanized by an overdose of pentobarbital (Esconarkon^®^, Streuli Tiergesundheit AG, Uznach, Switzerland) due to acute pneumonia on d0.

### 2.2 Assessment of fecal score and “rescue-treatment”

The fecal score was monitored for each pig on d −4, −1, 0, 1, 2, 3, 6 and 7, with d0 being the day of infection. To evaluate the fecal score, a cotton swab was introduced into piglet's rectum to collect a small amount of feces. The consistency of the feces was scored on a scale ranging from 1 to 4. Normal molted feces were given a score of 1 and watery diarrhea a score of 4. Liquid diarrhea was scored as 3, and creamy feces was given a score of 2. In addition, a VAS (Visual Analogic Score) was used in study 1 to assess the severity of the diarrhea. A VAS is a psychometric response scale used to measure subjective characteristics (29, 30). The VAS consisted of a 40-mm-long horizontal line with numeric descriptors (1–4) every 10 mm to represent fecal score class. An observer marked the point on the line that best reflects the observed severity of the diarrhea. Compared to fecal scores, VAS enabled the observer to characterize the severity of the diarrhea as a continuous process, thus capturing the quality of the feces in more detail. In study 1, feces were collected directly from the rectum of each pig to assess the Zn concentration on d −1, 0, 1, 2, 3, 6 and 7.

To ensure animal welfare, piglets with severe ETEC infection clinical signs, i.e., watery diarrhea for more than five consecutive days, or watery diarrhea and apathy, were removed from the study. These pigs are described as “rescue treated” as they were treated with AB (sulfamide trimethoprim, 15 mg/kg BW Borgal^®^ 24%, MSD AH, Switzerland) for five consecutive days to alleviate the clinical signs.

### 2.3 Assessment of intestinal inflammatory status and permeability of the intestinal mucosa

On the day of euthanasia, 100–200 mg of mucosa were scraped from the middle jejunum of piglets and immediately frozen in liquid nitrogen. Samples were kept at −80°C until further analysis. For IL-6 and IL-8 measurements, mucosa was homogenized by sonication in 20 mmol/L Tris base, 137 nmol/L NaCl, 1% NP-40 (Nonidet P-40) and 10% glycerol, supplemented with a cocktail of protease inhibitors (Complete TM, Sigma, Buchs, Switzerland). After 5 min of centrifugation at 800 g at 4°C, the supernatant was recovered and used to determine IL-6 and IL-8 concentrations using specific pig IL-6 and IL-8 ELISA kits (Abcam, Cambridge, United Kingdom) following the manufacturer's procedure. The results were expressed as ng of IL/mg of protein used in the assay.

The concentrations in tight junction proteins (i.e., occludin and claudin-1, claudin-5 and claudin-7) were assessed by western blots. The proteins were extracted by three freeze–thaw cycles from 100 to 200 mg of mucosa with CelLytic MT buffer (Sigma, Buchs, Switzerland), supplemented with a cocktail of protease inhibitors (Complete TM, Sigma, Buchs, Switzerland), using the freeze–thaw lysis method. Samples were then centrifuged for 10 min, 12,000 × g at 4°C. Proteins (20–30 μg) were separated on an 8% acrylamide SDS-PAGE gel and further transferred to polyvinylidene difluoride membranes (Fisher Scientific, Reinach, Switzerland), as described previously (31). Hybridizations were performed overnight at 4°C in PBS (phosphate buffered saline) supplemented with 0.1% Tween-20 and 5% BSA (bovine serum albumin). The following primary antibodies were used at the indicated dilutions: rabbit anti-occludin 1:1000 (Abcam, Cambridge, United Kingdom), rabbit anti-claudin-1 1:1000 (Abcam, Cambridge, United Kingdom), rabbit anti-claudin-7 1:500 (Fisher Scientific, Reinach, Switzerland) and mouse anti-claudin-5 1:200 (Fisher Scientific, Reinach, Switzerland). After washing, blots were incubated with horseradish peroxidase (HRP)-conjugated goat anti-rabbit or mouse IgG secondary antibodies diluted 1:1000 in PBS supplemented with 0.1% Tween-20 and 5% non-fat milk for 1 h at room temperature. Protein bands were further detected using chemiluminescence with a Quantum kit (Witec, Luzern, Switzerland). The intensity of the tight junction proteins signals was normalized to the signal of vinculin.

### 2.4 Assessment of systemic oxidative status

Piglets' oxidative status was determined in serum using commercial kits as instructed by the manufacturer. Serum was obtained from blood directly sampled during bleeding on the day of euthanasia. Blood was collected using blood collection tubes with serum clot activator (Vacuette; Greiner Bio-One GmbH), which were stored upside down at room temperature for 1 h before processing. The Vacuette serum tubes were then centrifuged for 15 min at 3,000 g and subsequently for 2 min at 4,000 g. Two aliquots of serum were stored at −20°C in Eppendorf tubes. The MDA equivalent content was assessed using the Lipid Peroxydation Assay kit (Sigma, Buchs, Switzerland). Total antioxidant status (TAS) was assessed using the Antioxidant Assay kit (Sigma, Buchs, Switzerland). Superoxide dismutase (SOD) activity was assessed using the Superoxide Dismutase colorimetric activity kit (Invitrogen, Waltham MA, USA). Finally, lactate dehydrogenase (LDH) activity was assessed using the human LDH SCE Humazym test (Human Diagnostics, Wiesbaden, Deutschland).

### 2.5 Analysis of the fecal ETEC excretion and the antibiotic resistome

The infective strain was resistant to rifampicine (rif) and harbored the F4 fimbriae (K88ac+), the heat-labile toxin (LT+) and the heat-stable toxin (STb+), as determined by PCR (11). The amount of F4 ETEC excreted was measured by counting the number of colony-forming units per culture on an *E. coli*-selective medium (Eosin Methylene Blue Agar plates) containing rifampin (50 μg/mL).

Measurements were performed on feces samples collected on d0, 1, 2, 3 and 6. On the day of euthanasia, the contents of the small intestine were collected from 6 pigs per group in study 1 and subjected to resistome analysis. qPCR was performed on 89 bacterial resistance genes (Qiagen microbial PCR array, Qiagen AG, Hombrechtikon, Switzerland) following the manufacturer's recommendations. In brief, DNA was extracted from each sample (QIAamp kit, Qiagen AG, Hombrechtikon, Switzerland) and mixed with MicrobialqPCR mastermix (included in Qiagen microbial PCR array kit) and then aliquoted to each well of the plate containing a pre-dispensed gene-specific primer and hydrolysis probe set. The array can simultaneously target a broad-spectrum profile of 89 genes from all major classes of antibioresistance genes (Supplementary Table S2), including aminoglycoside, β-lactam, erythromycin, quinolone and fluoroquinolone, macrolide-lincosamide-streptogramin_b, tetracycline and vancomycin.

### 2.6 Chemical analysis of the diets, Zn concentrations in diets and feces

Each diet was analyzed in triplicate to determine its chemical composition, including crude protein, crude fiber and fat content. Feces collected on d0, 1, 2, 3 and 6 (study 1) were pooled together, homogenized and analyzed for Zn to compare the excretion of Zn relative to the quantity in the diet in the pZnO-150, pZnO-300, C and C-3000 groups. In brief, all samples were freeze-dried before analyses (Christ Delta 2-24, Kühner AG, Birsfelden, Switzerland). After being ground to pass a 1-mm screen (Brabender rotary mill; Brabender GmbH & Co. KG, Duisburg, Germany), feed samples were analyzed for dry matter content by heating at 105°C for 3 h followed by incineration at 550°C until a stable mass was reached to determine the ash content according to ISO 5984_2002 (prepASH, Precisa Gravimetrics AG, Dietikon, Switzerland).

The tannin content of the tannin-rich extract was determined by the ISO 14088 method. Zn content was analyzed according to EN 15510:2008 by ICP-OES (ICP-OES 5800, Agilent Technologies, Switzerland) after microwave digestion. Samples were dissolved in a glass tube (5 mL HNO3 65% + 3 mL H2O ASTM Class I) using a microwave digester (UltraClave MLS, Leutkirch, Germany) at 235°C for 60 min (1000 W). If necessary, samples were diluted with HNO3 2% prior to analysis.

### 2.7 Statistical analyses

All analyses were performed with R studio (v3.6.3) using the nlme, MASS, FSA, ordinal, car, emmeans and multicomp packages. Fecal scores were analyzed with a repeated two-way ordinal regression, with dietary treatment, study day and farrowing series as fixed effects. All other variables were analyzed with a linear mixed effect model, with dietary treatment, study day (if appropriate) and farrowing series as fixed effects. Interactions between dietary treatment, study day and farrowing series were always tested and only retained in the model if they were significant. In general, the models were reduced by stepwise exclusion of non-significant (*P* > 0.05) interactions and factors (except dietary treatment and study day). Least-squares means of the response variables and Tukey–Kramer pairwise comparisons were computed if the dietary treatment was significant, and differences were considered significant if *P* < 0.05 (a tendency was considered if *P* < 0.10). Homoscedasticity and normality of the residues were checked for each model. In case of interaction, the contrast of the parameter of interest was always calculated for fixed values of the interactive parameter using the emmeans package.

Feed intake was calculated per day as the amount of feed disappearance in the pen and divided by the number of animals, and the pen was used as the experimental unit. Average daily feed intake (ADFI) was calculated as the average daily feed intake of a pen for a specific period. Diarrhea was defined as a fecal score above 2, and the duration of diarrhea was calculated as the sum of days in diarrhea for each pig. The daily prevalence of diarrhea was calculated as the percentage of pigs in a dietary treatment having a fecal score greater than 2. Feed efficiency was calculated as the sum of total feed used per pen divided by the total weight gain of the pen.

For all animals removed from the study, missing values were attributed to all subsequent measurements after the removal date. Exceptions were made for “rescue-treated” animals, where the last fecal score and VAS measured were carried over to all subsequent measurements, to penalize ineffective treatment, as done in clinical trials for drug development (32).

## 3 Results

### 3.1 Severity of diarrhea and number of pigs “rescue treated”

A total of 12 pigs were “rescue treated” with AB due to severe ETEC infection symptoms. Nine pigs were in the C group, with five and four pigs in studies 1 and 2, respectively. In contrast, the other treatment groups (pZnO-150, pZnO-300, C-3000, TAN and TAN + pZnO-150) each had either one pig or no pigs that needed rescue treatment, *p* = 0.05 (Table 1).

**Table 1:** Number of pigs rescue treated, duration and daily prevalence of diarrhea.

**Study 1** ^**1**^						
**Parameters**	**C**	**C-3000**	**pZnO-150**	**pZnO-300**	**SEM**	***p*** **-value** ^2^
Number of pigs rescue treated^3^	5	1	0	1	NA	0.05
Average duration of diarrhea, d	5.3^a^	1.6^b^	4.8^a^	4.7^a^	0.40	< 0.0001
Average daily prevalence of diarrhea, %	55^a^	19^b^	59^a^	51^a^	54.7	0.001
**Study 2** ^4^						
	**C**	**TAN**	**pZnO-150**	**TAN**+**pZnO-150**	**SEM**	***p*** **-value**
Number of pigs rescue treated	4	0	1	0	NA	0.05
Average duration of diarrhea, d	5.5	4.9	4.9	4.7	0.29	-
Average daily prevalence of diarrhea, %	85	75	78	73	78.8	-

To ensure animal welfare, piglets with severe ETEC infection symptoms, i.e., watery diarrhea for more than five consecutive days, or watery diarrhea and apathy, were removed from the study and qualified as rescue treated. Fecal score was evaluated on a scale of 1–4 on days −4, −1, 0, 1, 2, 3, 6 and 7. Normal molted feces were given a score of 1, while watery diarrhea was given a score of 4. Daily prevalence of diarrhea corresponds to the percentage of piglets within the experimental treatment with a fecal score >2.

^1^C: control group with standard starter diet and 150 mg/kg Zn of conventional ZnO (maximal Zn concentration allowed in EU); C-3000: positive control group with standard starter diet with 3,000 mg/kg Zn of conventional ZnO; pZnO-150 and pZnO-300: starter diet with 150 and 300 mg/kg Zn of potentiated ZnO, respectively.

^2^*p*-value: The *p*-value reported here corresponds to the pairwise comparison of values.

^3^Number of piglets treated with antibiotics due to severe ETEC infection symptoms.

^4^C: control group with standard starter diet and 150 mg/kg Zn of conventional ZnO; TAN: starter diet with 7.5 g/kg of tannin extract and 150 mg/kg Zn of conventional ZnO; TAN+pZnO-150: starter diet with 7.5 g/kg of tannin extract and 150 mg/kg Zn of potentiated ZnO; pZnO-150: starter diet with 150 mg/kg Zn of potentiated ZnO.

^a, b^Different letters indicate significant (*p* < 0.05) differences between the values.

^*, **^Difference in the number of stars indicate a difference for which the *p*-value is between 0.05 and 0.10.

**Figure 1:**
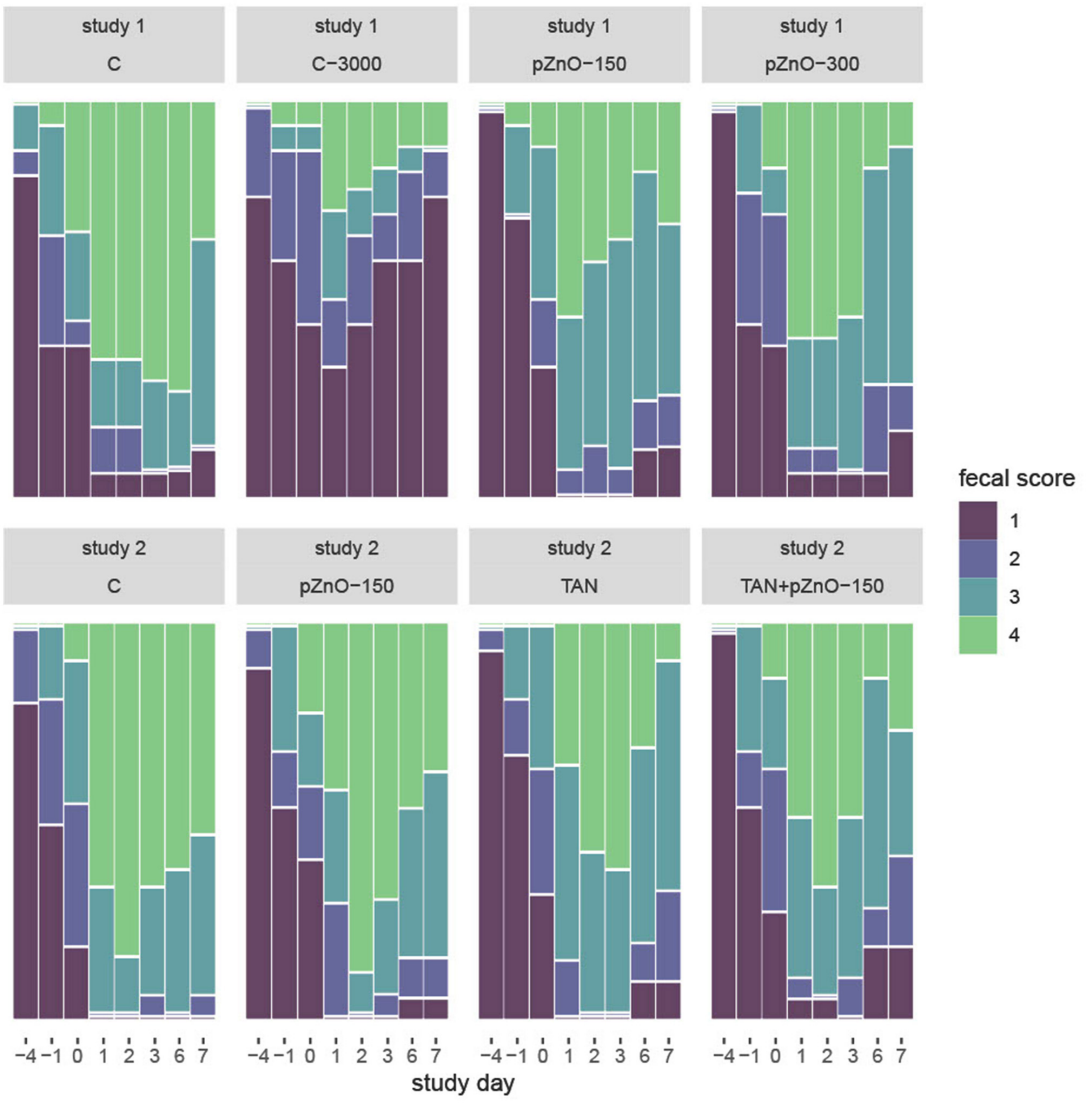
Mosaic plot of fecal score in relation to study day in studies 1 and 2. Fecal score was evaluated on a scale of 1–4. Normal molted feces were given a score of 1, watery diarrhea a score of 4. C: control group with standard starter diet and 150 mg/kg Zn of conventional ZnO (maximal Zn concentration allowed in EU); C-3000: positive control group with standard starter diet with 3,000 mg/kg Zn of conventional ZnO; pZnO-150 and pZnO-300: starter diet with 150 and 300 mg/kg Zn of potentiated ZnO, respectively; TAN: starter diet with 7.5 g/kg of tannins extract and 150 mg/kg Zn of conventional ZnO; TAN+pZnO-150: starter diet with 7.5 g/kg of tannins extract and 150 mg/kg Zn of potentiated ZnO.

Infection on d0 led to an increased fecal score in each dietary treatment on d1 (*p* < 0.0001), except for C-3000 (Figure 1). In study 1, group C had higher fecal scores than group C-3000 on d-1, 0, 1, 2, 3, 6 and 7 (*p* < 0.0001). Regardless of the day, there was no difference in fecal scores between C and the two other experimental treatments (pZnO-150 and pZnO-300). Starting on d1, pigs in the C-3000 group had lower fecal scores than those in the pZnO-150 or pZnO-300 groups (*p* < 0.01). The visual analogic score (VAS) followed the same pattern as the fecal scores (Supplementary Figure S1). In study 2, TAN and TAN+pZnO-150 pigs had lower fecal scores than C starting on d1 (*p* < 0.05).

The duration of diarrhea was 1.6 days on average in group C-3000 and over 4 days in the C, pZnO-150 and pZnO-300 groups (*p* < 0.0001; Table 1). The average daily prevalence of diarrhea, expressed as the percent of pigs with diarrhea within the experimental treatment, was 19% in C-3000 compared to > 50% in the other groups in study 1 (*p* < 0.001). In study 2, there was no effect of the groups on the duration (5 days, on average) or daily prevalence of diarrhea (>72% in all groups).

### 3.2 Growth performances

**Figure 2:**
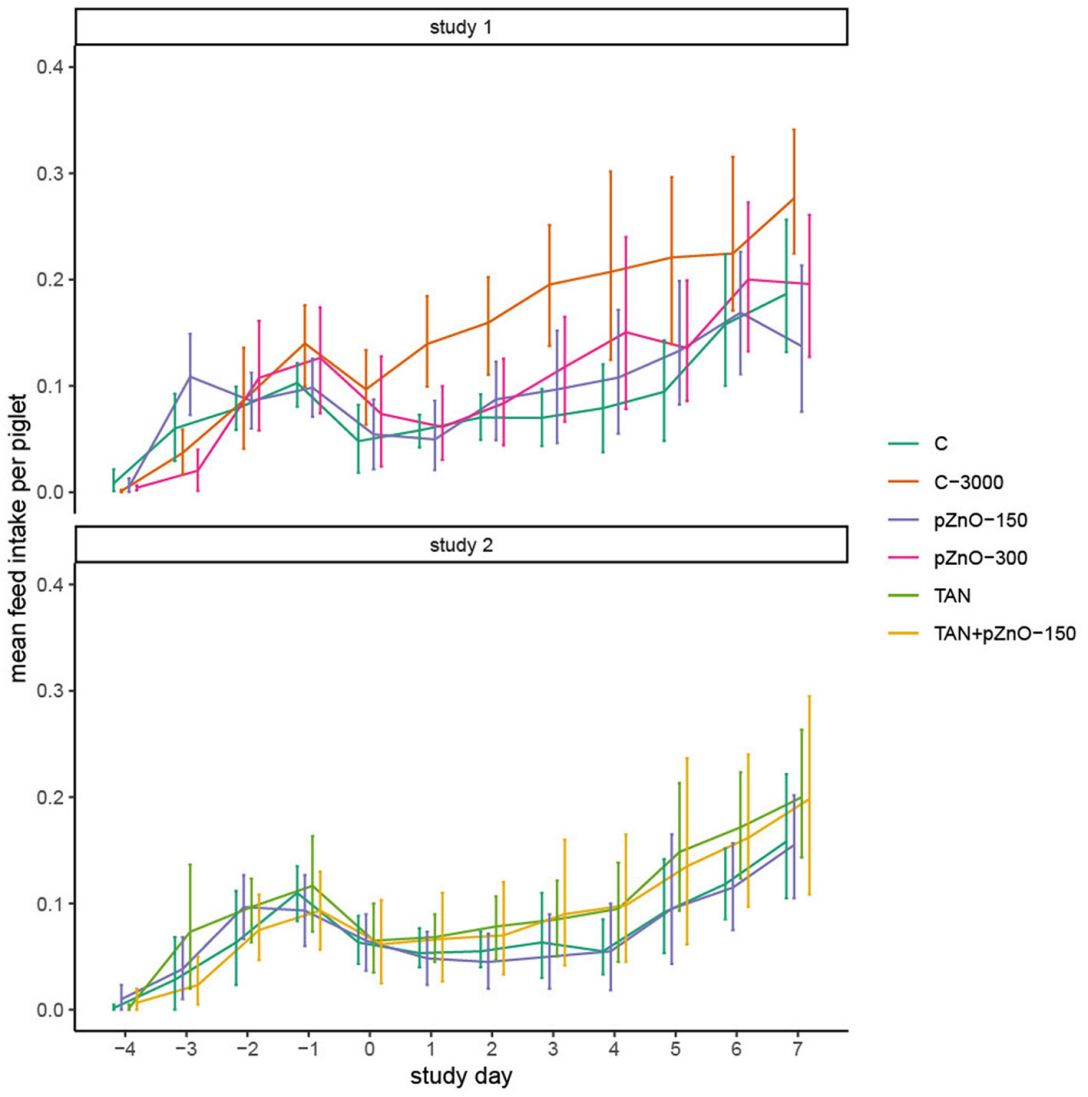
Mean daily feed intake calculated per piglet and per pen. The daily feed intake was measured per pen and divided by the number of pigs in the pen, as the number of pigs per pen was not always the same (mortality). The pigs ate little on weaning day, and their consumption increased slightly until d-1, before dropping again after infection.

In study 1, C-3000 pigs grew faster (*p* < 0.05) from d4 to d7 than pigs in the other experimental treatments, which resulted in a greater bodyweight on day 7 (*p* = 0.02; Table 2). Improved growth was associated with a greater average daily feed intake (ADFI) after ETEC infection (Figure 2; *p* < 0.05). There was a significant interaction between experimental treatments and time for the variables BW and ADFI. In study 2, BW, average daily gain (ADG), ADFI and feed efficiency (FE) were not affected by the dietary treatments (Table 2). Regardless of the study, pigs ingested only a small amount of feed the day of weaning (d −4), and the consumption slightly increased until d −1 before dropping again for all dietary treatments after infection at d0. In study 1, the C-3000 pigs ate 0.10 kg more on d7 than the rest of the pigs (*p* < 0.01). In study 2, the feed consumption on d7 in dietary treatments TAN and TAN+pZnO-150 was 0.05 kg higher than in the other dietary treatments (p>0.05). There was no difference in FE among the dietary treatments.

**Table 2:** BW, ADG, ADFI, and FE.

	**Days**	**Study 1 (*****n*** = **18 pigs/group)**						**Study 2 (*****n*** = **22 pigs/group)**					
		**Treatments** ^1^						**Treatments** ^2^					
		**C**	**C-3000**	**pZnO-150**	**pZnO-300**	**SEM**	***p*** **-value** ^3^	**C**	**TAN**	**pZnO-150**	**TAN**+**pZnO-150**	**SEM**	***p*** **-value**
BW^4^, kg	−4	7.74	7.62	7.95	7.75	0.246	-	7.74	7.53	7.61	7.60	0.217	-
	0	7.61	7.91	7.91	7.72	0.234	-	7.59	7.57	7.53	7.61	0.207	-
	7	7.38^a^	8.42^b^	7.84^a^	7.67^a^	0.256	0.02	7.33	7.64	7.39	7.62	0.218	-
ADG^5^, g/d	0–7	52	80	36	21	89.0	-	−23	19	4	31	23.8	-
	−4 to 7	−18^a^	80^b^	−35^a^	−11^a^	27.2	0.05	−37	10	−28	−1	16.8	-
	−4 to 0	−125	−59	−119	−80	26.0	-	−53	−4	−54	−46	18.4	-
ADFI^6^, g/d	0–7	102^a^	214^b*^	118^a, b**^	135^a, b^	31.6	0.05	82	114	78	110	30.1	-
	−4 to 7	83^*^	154^**^	98	106	25.3	-	72	100	72	90	23.3	-
	−4 to 0	57	72	70	66	17.9	-	53	70	61	52	12.3	-
FE, g/g^7^	0–7	0.6	0.1	0.0	0.2	0.50	-	−0.6	0.1	−0.3	−0.4	1.16	-
	−4 to 7	−0.1	0.4^*^	−0.7^**^	−0.3	0.29	-	−0.9	0.0	−0.5	−0.5	0.41	-
	−4 to 0	−3.6	−1.5	−2.4	−2.9	1.19	-	−1.5	−0.1	−1.2	−1.3	0.56	-

^1^C: control group with standard starter diet and 150 mg/kg Zn of conventional ZnO (maximal Zn concentration allowed in EU); C-3000: positive control group with standard starter diet with 3,000 mg/kg Zn of conventional ZnO; pZnO-150 and pZnO-300: starter diet with 150 and 300 mg/kg Zn of potentiated ZnO, respectively.

^2^C: control group with standard starter diet and 150 mg/kg Zn of conventional ZnO (maximal Zn concentration allowed in EU); TAN: starter diet with 0.75% of tannin extract and 150 mg/kg Zn of conventional ZnO; TAN+pZnO-150: starter diet with 0.75% of tannin extract and 150 mg/kg Zn of potentiated ZnO; pZnO-150: starter diet with 150 mg/kg Zn of potentiated ZnO.

^3^*p*-value: The *p*-value reported here corresponds to the pairwise comparison of values.

^4^BW, Bodyweight.

^5^ADG, Average daily gain.

^6^ADFI, Average daily feed intake.

^7^FE, Feed efficiency.

^a, b^Different letters indicate significant (*p* < 0.05) differences between the values.

^*, **^Difference in the number of stars indicates a difference for which the *p*-value is between 0.05 and 0.10.

### 3.3 Inflammatory and health status of the small intestine

**Figure 3:**
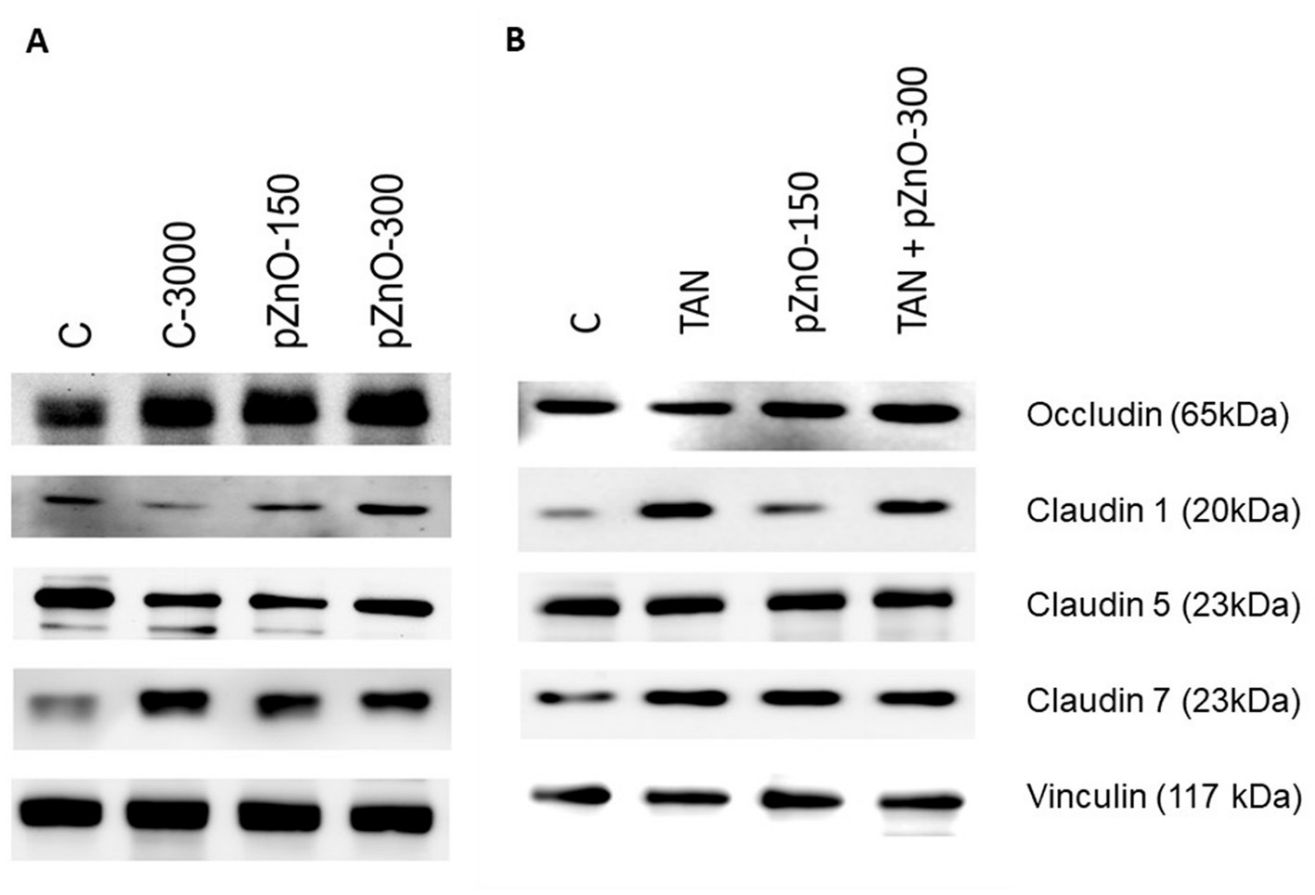
Representative protein bands for the tight junction proteins. Piglets were euthanized on d9 (±1), and small intestine mucosa was scraped for western blot (tight junction proteins) and interleukin analyses. **(A)** study 1: C: control group with standard starter diet and 150 mg/kg Zn of conventional ZnO (maximal Zn concentration allowed in EU); C-3000: positive control group with standard starter diet with 3,000 mg/kg Zn of conventional ZnO; pZnO-150 and pZnO-300: starter diet with 150 and 300 mg/kg Zn of potentiated ZnO, respectively; **(B)** study 2: TAN: starter diet with 7.5 g/kg of tannins extract and 150 mg/kg Zn of conventional ZnO; TAN+pZnO-150: starter diet with 7.5 g/kg of tannins extract and 150 mg/kg Zn of potentiated ZnO.

In both studies, the concentrations of tight junction proteins occludin, claudin 1 and claudin 5 in the small intestine were not affected by the dietary treatments (Figure 3; Table 3). In contrast, the concentration of claudin 7 was greater in C-3000 pigs than in pZnO-150 pigs in study 1, and it was greater in pZnO-150 and TAN pigs than in TAN + pZnO-150 pigs in study 2. The concentrations of interleukin 6 (IL-6) and interleukin 8 (IL-8) were not affected by the dietary treatment in either study (Table 3). In study 2, the Malondialdehyde (MDA) concentrations were higher in the TAN than in the C and pZnO-150 groups (*p* < 0.05; Table 3). In the pZnO-150 group, TAS concentrations were lower than in the TAN + pZnO-150 group (*p* < 0.05).

**Table 3:** Inflammatory and health status of the small intestine.

**Study 1**^**1**^ **(*****n*** = **18 pigs/group)**						
**Parameters (unit)**	**C**	**C-3000**	**pZnO-150**	**pZnO-300**	**SEM**	***p*** **-value** ^2^
**Small intestine mucosal concentrations**						
Occludin (RU^3^)	0.36	0.25	0.52	0.30	0.140	-
Claudin 1 (RU)	0.40	0.36	0.48	0.53	0.141	-
Claudin 5 (RU)	4.2	0.8	1.4	1.2	1.52	-
Claudin 7 (RU)	0.39	0.70^a^	0.34^b^	0.35	0.083	0.04
IL8^4^ (pg/mg)	558	451	607	602	59.1	-
IL6^5^ (pg/mg)	26.2	19.7	19.2	25.2	4.46	-
**Study 2**^6^ **(*****n*** = **22 pigs/group)**						
	**C**	**TAN**	**pZnO-150**	**TAN**+**pZnO-150**	**SEM**	***p*** **-value**
**Small intestine mucosal concentrations**						
Occludin (RU)	0.35	0.26	0.19	0.37	0.062	-
Claudin 1 (RU)	0.11^*^	0.15^*^	0.18^*^	0.28^**^	0.053	0.06
Claudin 5 (RU)	0.9	1.3	1.7	1.7	0.45	-
Claudin 7 (RU)	0.76	1.00^a^	1.12^a^	0.59^b^	0.105	0.03
IL8 (pg/mg)	424	383	414	414	50.0	-
IL6 (pg/mg)	7.5	9.5	7.0	6.7	1.09	-
**Serum concentrations**						
MDA^7^ (mmol/L)	16.7^a^	19.8^b^	16.9^a^	17.1	0.79	0.03
TAS^8^ (mmol/L)	0.36	0.34	0.32^a^	0.36^b^	0.011	0.03
SOD^9^ (U/mL)	1.2	1.2	1.2	1.4	0.08	-
LDH^10^ (U/L)	849	1062	902	938	65.1	-

Piglets were euthanized on d9 (±1), and small intestine mucosa was scraped for western blot (tight junction proteins) and interleukin analyses. Blood sampling was also performed to assess oxidative status in study 2.

^1^C: control group with standard starter diet and 150 mg/kg Zn of conventional ZnO (maximal Zn concentration allowed in EU); C-3000: positive control group with standard starter diet with 3,000 mg/kg Zn of conventional ZnO; pZnO-150 and pZnO-300: starter diet with 150 and 300 mg/kg Zn of potentiated ZnO, respectively.

^2^*p*-value: The *p*-value reported here corresponds to the pairwise comparison of values.

^3^RU, relative unit; The intensity of the tight junction protein western blot signals was normalized to the signal of vinculin.

^4^IL8, interleukin 8; expressed in pg/mg of total proteins.

^5^IL6, interleukin 6; expressed in pg/mg of total proteins.

^6^C: control group with standard starter diet and 150 mg/kg Zn of conventional ZnO (maximal Zn concentration allowed in EU); TAN: starter diet with 0.75% of tannin extract and 150 mg/kg Zn of conventional ZnO; TAN+pZnO-150: starter diet with 0.75% of tannin extract and 150 mg/kg Zn of potentiated ZnO; pZnO-150: starter diet with 150 mg/kg Zn of potentiated ZnO.

^7^MDA, Malondialdehyde.

^8^TAS, Total antioxidant status.

^9^SOD, Super oxide dismutase.

^10^LDH, Serum lactate dehydrogenase.

^a, b^Different letters indicate significant (*p* < 0.05) differences between the values.

^*, **^Difference in the number of stars indicate a difference for which the *p*-value is between 0.05 and 0.10.

### 3.4 Zinc and ETEC excretion, resistome

The C-3000 pigs excreted 12,312 mg Zn/kg dry mater of feces, which was higher (*p* < 0.0001, Table 4) than the fecal Zn excretion in the C, pZnO-150 and pZnO-300 groups (1,028 mg Zn/kg dry mater of feces, on average). The excretion of ETEC F4 was the lowest in the C-3000 pigs and the highest in the pZnO-150 pigs (*p* < 0.05), with intermediate excretion levels for the pZnO-300 and C pigs in study 1 (Table 4). There was no dietary treatment effect on the ETEC F4 excretion level in study 2.

**Table 4:** Resistome, Zn and ETEC excretion.

**Study 1** ^**1**^						
**Parameters**	**C**	**C-3000**	**pZnO-150**	**pZnO-300**	**SEM**	***p*** **-value** ^2^
Zinc (mg/Kg DM feces)	626^a^	12312^b^	628^a^	1832^a^	483	< 0.001
ETEC CFU^3^ (10^4^ U), d7	18.2	3.0^a^	33.5^b^	19.5	31.29	0.02
Number of copies of resistance genes^4^	100.00^a^	1318.26^c^	0.02^b^	0.01^b^	157.761	< 0.001
**Study 2** ^5^						
	**C**	**TAN**	**pZnO-150**	**TAN**+**pZnO-150**	**SEM**	***p*** **-value**
ETEC CFU (10^4^ U), d7	2.1	4.0	7.8	2.1	3.71	-

Feces samples were collected on d0 (before infection), 1, 2, 3 and 6 for ETEC excretion measurement in colony forming unit (CFU). Feces samples were pooled to analyze the Zn content. Resistome analysis was performed on small intestine contents collected on d9 (±1) in study 1 only. The number of resistance genes was calculated relative to the C group and considering 100 resistance genes in the C group.

^1^C: control group with standard starter diet and 150 mg/kg Zn of conventional ZnO (maximal Zn concentration allowed in EU); C-3000: positive control group with standard starter diet with 3,000 mg/kg Zn of conventional ZnO; pZnO-150 and pZnO-300: starter diet with 150 and 300 mg/kg Zn of potentiated ZnO, respectively.

^2^*p*-value: The *p*-value reported here corresponds to the pairwise comparison of values.

^3^The number of colony-forming units (CFUs) was assessed by g of feces in study 1 and by the quantity collected by a swab in study 2.

^4^Number of resistance genes: Number of resistance genes calculated considering 100 resistance genes in the C group.

^5^C: control group with standard starter diet and 150 mg/kg Zn of conventional ZnO (maximal Zn concentration allowed in EU); TAN: starter diet with 0.75% of tannin extract and 150 mg/kg Zn of conventional ZnO; TAN+pZnO-150: starter diet with 0.75% of tannin extract and 150 mg/kg Zn of potentiated ZnO; pZnO-150: starter diet with 150 mg/kg Zn of potentiated ZnO.

^a, b, c^Different letters indicate significant (*p* < 0.05) differences between the values.

In study 1, C-3000 dietary treatment led to a 13-fold increase in the number of resistance genes compared to C-group (*p* < 0.001), whereas pZnO-150 and pZnO-300 resulted in a net decrease (*p* < 0.001; Table 4).

## 4 Discussion

The high dose of ZnO led to a reduction of AB treatment, lower fecal score and increased feed intake, resulting in higher ADG but not higher FE. The high dose of ZnO also resulted in a high Zn excretion and a greater number of copies of resistance genes in the intestinal microbiota compared to the control group. The potentiated source of ZnO in the 150 and 300 mg Zn/kg diets reduced the severity of infection, leading to a lower number of AB treatments. A mix of hydrolysable and condensed tannin extract given at 7.5 g/kg diet with or without a pZnO (150 mg Zn/kg) also contributed to reducing the fecal score and the number of AB treatments, without affecting feed intake, ADG or FE.

The effects of pZnO on the ADG and feed intake of post-weaning pigs vary across studies. Compared to the current study, Peng et al. (17) and Wang et al. (15) reported that starter diets supplemented with 200–500 mg/kg pZnO increased the ADG and feed intake of weaned piglets that were not subjected to an ETEC challenge. Trevisi et al. (18) reported that supplementing starter diets with 150–300 mg/kg pZnO increased average daily gain (ADG) in ETEC-challenged F18/4-susceptible piglets within 2 weeks after weaning, though the increase in feed intake was not statistically significant. Compared to the present study, Trevisi et al. (18) used a markedly lower infection load of 10^7^ ETEC. This might explain the difference in the impact of the pZnO on the growth performance traits. Additionally, in the present study, feed intake was not measured at the individual level but rather calculated as the average individual feed intake per pen. This pen-level assessment may be less accurate than individual measurements.

There was no reduction in the prevalence of diarrhea using a low dose of pZnO in the present study, which is in agreement with Wang et al. (15), who reported no reduction in the prevalence of diarrhea when pZnO was added to a starter diet at 110 or 220 mg/kg. On the contrary, in the study of Peng et al. (17), the prevalence of diarrhea was reduced by more than 4% (*p* < 0.01) when piglets were fed a diet supplemented with 200 or 500 mg/kg pZnO.

The high dose of ZnO performed as expected (17, 18, 33, 34): diarrhea severity reduced, and the piglets became healthier, ate more and grew faster. Therefore, the present model was validated *per se*, with a positive control group performing much better than the negative control group.

The effects of tannins in the present study are consistent with the analysis of Caprarulo et al. (19), who reported that tannins given in low doses (<1%) did not significantly improve animals' overall performances traits after weaning, although their growth performance tended to improve numerically. Indeed, quebracho tannins given at doses up to 0.3% had no clear effect on ADG, feed intake or FE, but they reduced fecal scores when the piglets were weaned without artificial infection (25). Even at the 1% inclusion level, chestnut tannin extract improves only numerically ADG and feed intake when tested using the same in-house infection model (23), but it did reduce the severity and duration of diarrhea. It is only when given at 2% inclusion level that chestnut extract markedly increased weight gain and feed intake while limiting the severity and duration of diarrhea (22).

Although some performances were numerically improved, there was no evidence of synergies between tannins and pZnO relative to feed intake, ADG or fecal score. These results are consistent with the observations of Liu et al. (35), who tested a high dose of ZnO (2,000 mg/kg Zn, conventional source) with or without the addition 0.1% of tannin extract (containing 0.75% of chestnut tannins) in a conventional weaning process, namely without artificial infection. Only the diarrhea incidence rate was reduced by ZnO and tannins, but the combination of the two did not have greater efficacy than either treatment separately.

The source and dose of zinc, with or without tannins, had very little impact on the inflammation status of the piglets, as demonstrated by the absence of changes in IL-6 and IL-8 levels compared to the control group. However, tissues for IL-6 and IL-8 measurements were sampled 9 (±1) days after infection, which may be too late to detect acute inflammation. The effect of ZnO on the inflammation status of pigs remains controversial. Indeed, results of *in vitro* studies (36, 37) tend to indicate that ZnO reduces the inflammatory response by reducing the IL-8 and tumor necrosis factor alpha (TNF-α) serum levels of ETEC-infected intestinal cells. In contrast, the results of *in vivo* studies are less consistent. Pei et al. (34) reported an increase in IL-6 and TNFα serum levels in non-infected pigs fed with nano ZnO (150 and 300 mg/kg) or with a high dose of conventional ZnO (3,000 mg/kg) when compared to a basal diet. In agreement with the present results, Wang et al. (15) found that neither the source (pZnO or ZnO) nor the inclusion level (pZnO at 110 or 220 mg/kg Zn or ZnO at 2,400 mg/kg Zn) affected the mRNA levels of pro-inflammatory cytokines like TNF-α, interferon gamma and interleukin-1 beta in piglets weaned without artificial infection.

Improvements in the intestinal barrier due to ZnO administration have been reported in several studies: high doses of ZnO (>2,000 mg/kg Zn) increased the gene expression of occludin and claudin 1 (15, 38) and protein levels of occludin (38) in conventionally weaned piglets. In the present study, an increase in claudin 7 intestinal protein expression was only observed in C-3000 pigs. In study 2, TAN+pZnO pigs had higher claudin 1 intestinal protein expression levels than the pigs in the other treatment groups, but they had lower claudin 7 intestinal protein expression than TAN and pZnO-150 pigs. As previously described, both TAN (39) and ZnO (15, 38) enhance tight junction protein expression. However, the low dose of TAN (0.75%) and pZnO (150 mg/kg Zn), alone, may not have been sufficient to trigger greater protein expression of the tight junction proteins.

TAN and pZnO inconsistently affected the oxidative status of the animals in this study. The MDA levels were higher in the TAN groups than in the C-150 and pZnO-150 groups, indicating higher oxidative stress, whereas tannins are known for their antioxidative properties (35, 40). In addition, the total antioxidant status was lower in the pZnO group than in the TAN+pZnO group, which is not consistent with the aforementioned results. These inconsistencies make it difficult to interpret those results.

High doses of ZnO have been shown to increase the occurrence of zinc-resistant bacteria in the pig gut microbiota and to play a significant role in the co-selection of methicillin-resistant *Staphylococcus aureus* (MRSA) (41). Recent studies have suggested that feeding ZnO at high concentrations during weaning increases the proportion of multidrug-resistant *E. coli* in the gut of the piglets (11, 42). We also observed an increase in the number of copies of antimicrobial resistance genes in the C-3000 group compared to the C group. However, our approach aimed at detecting resistance genes in all bacteria from the small intestine rather than in one specific bacteria. The exact reasons for this co-selection of antibiotic resistance genes are unknown. Possible mechanisms include physiological coupling, such as the interaction of heavy metals with efflux pumps, and genetic coupling, such as co-resistance or the association of antimicrobial and heavy metal resistance genes on mobile genetic elements (41). The interaction of heavy metals with the bacterial conjugation system is another possible mechanism of action that is more supported by recent studies (10, 11, 42). Unfortunately, the present study did not investigate the heavy metal resistance genes, which limits the interpretation of the present results. Bacteria under stress may also have a higher conjugation rate, which would facilitate the exchange of genetic material that contains antimicrobial resistance genes (10).

The pharmacological dose of ZnO (2,500 mg Zn/kg diet) improved growth performance traits and alleviated PWD symptoms but increased Zn excretion and antimicrobial resistance genes. Tannin-rich extract and pZnO alleviated PWD, leading to a decrease in AB usage. Furthermore, tannins reduced the fecal scores.

## Data Availability

The raw data supporting the conclusions of this article will be made available by the authors, without undue reservation.
