# Supplementary material for: Feed supplementation with potentiated zinc and/or tannin-rich extracts reduces ETEC infection severity and antimicrobial resistance genes in pig
Source: Front Vet Sci. 2025 Feb 21;12:1494103. doi: 10.3389/fvets.2025.1494103 (PMC11887510; doi:10.3389/fvets.2025.1494103)
Supplement: Supplementary file 1 [file Table_1.docx]

Supplementary Material

Feed supplementation with potentiated zinc and/or tannin-rich extracts impact ETEC infection severity and antimicrobial resistance genes in pigs.

Catherine Ollagnier^*^, Johana Bellon, Maria-Rita Mellino, Nicolas Pradervand, Marco Tretola, Sebastien Dubois, Agathe Romeo, Olivier Desrues, Giuseppe Bee

# * Correspondence: Corresponding Author: [catherine.ollagnier@agroscope.admin.ch](mailto:catherine.ollagnier@agroscope.admin.ch)

**S.1 Nutrient, digestible energy, tannins and Zn content of the experimental diets.**

Table S.1: Nutrient, digestible energy, tannins and Zn content of the experimental diets. Each diet was formulated according to the Swiss feeding recommendations for weaned pigs [37] and was analysed in triplicate to determine the gross chemical composition.

|  | **Study 1** | | | | **Study 2** | | | |
| --- | --- | --- | --- | --- | --- | --- | --- | --- |
| **Parameters (unit)** | **C** | **C-3000** | **pZnO-150** | **pZnO-300** | **C** | **TAN** | **pZnO-150** | **TAN+**  **pZnO-150** |
| Dry matter (g/kg) | 903.4 | 904.1 | 904.2 | 901.7 | 887.7 | 891.1 | 885.5 | 887.2 |
| Crude protein^[[1]](#endnote-1)^ (g/kg) | 183.5 | 185.0 | 184.0 | 171.0 | 173.0 | 174.0 | 177.7 | 171.1 |
| Crude fat (g/kg)^[[2]](#endnote-2)^ | 31.0 | 30.6 | 30.3 | 30.7 | 30.5 | 31.2 | 28.2 | 31.3 |
| Crude fiber^[[3]](#endnote-3)^ (g/kg) | 42.8 | 38.8 | 43.6 | 38.8 | 40.2 | 40.6 | 43.2 | 39.4 |
| Digestible energy^[[4]](#endnote-4)^ (MJ/kg) | 13.6 | 13.6 | 13.6 | 13.6 | 13.6 | 13.6 | 13.6 | 13.6 |
| Tannin extract  (% DM) | - | - | - | - | - | 0.75 | - | 0.75 |
| Zn (g/kg DM) | 166.8 | 3237.6 | 155.2 | 348.6 | 199.6 | 189.9 | 167.2 | 168.7 |

1. Nitrogen content was analyzed with the Dumas method (ISO 16634–1:2008) and a multiplicative coefficient of 6.25 was applied to calculate crude protein content. [↑](#endnote-ref-1)
2. Fat content was extracted with petrol ether after an acid hydrolysis (ISO 6492:1999) [↑](#endnote-ref-2)
3. Crude fiber content was determined gravimetrically (ISO 6865:2000) by incineration of residual ash after acid and alkaline digestions using a fiber analyzer (Fibretherm Gerhard FT-12, C. Gerhardt GmbH & Co. KG, Koenigswinter, Germany) [↑](#endnote-ref-3)
4. The digestible energy coefficients from each feed ingredient were obtained from the Swiss Feed Database (https://www.feedbase.ch), and, taking into account the relative amount of each feed ingredient in the diet, the digestible energy content was calculated. [↑](#endnote-ref-4)
