# Supplementary material for: Feed supplementation with potentiated zinc and/or tannin-rich extracts reduces ETEC infection severity and antimicrobial resistance genes in pig
Source: Front Vet Sci. 2025 Feb 21;12:1494103. doi: 10.3389/fvets.2025.1494103 (PMC11887510; doi:10.3389/fvets.2025.1494103)
Supplement: Supplementary file 2 [file Table_2.docx]

Supplementary Material

Feed supplementation with potentiated zinc and/or tannin-rich extracts impact ETEC infection severity and antimicrobial resistance genes in pigs.

Catherine Ollagnier^*^, Johana Bellon, Maria-Rita Mellino, Nicolas Pradervand, Marco Tretola, Sebastien Dubois, Agathe Romeo, Olivier Desrues, Giuseppe Bee

# * Correspondence: Corresponding Author: [catherine.ollagnier@agroscope.admin.ch](mailto:catherine.ollagnier@agroscope.admin.ch)

**S.2 Resistance genes**

**Table S2:** A qPCR on 89 bacterial resistance genes (Qiagen microbial PCR array, Qiagen AG, Hombrechtikon, Switzerland) was performed according to manufacturer recommendations on the small intestine content (resistome) sampled on the day of euthanasia. In brief, DNA was extract from each sample (QIAamp kit, Qiagen AG, Hombrechtikon, Switzerland) and mixed with MicrobialqPCR mastermix (included in Qiagen microbial PCR array kit) and then aliquoted to each well of the plate containing the pre-dispensed gene-specific primer and hydrolysis probe set. Four PCR reactions were set up for each sample. The array can simultaneously target a broad-spectrum profile of 89 genes from all major classes of antibioresistance genes.

| **Species/Gene** | **Antibiotic classification/Virulence Factor Gene Description** |
| --- | --- |
| AAC(6)-Ib-cr | Fluoroquinolone resistance |
| aacC1, aacC2, aacC4, aadA1, aphA6 | Aminoglycoside resistance |
| BES-1, BIC-1, CTX-M-1 Group, CTX-M-8 Group, CTX-M-9 Group, GES, IMI & NMC-A, KPC, Per-1 group, Per-2 group, SFC-1, SFO-1, SHV, SHV(156D), SHV(156G), SHV(238G240E), SHV(238G240K), SHV(238S240E), SHV(238S240K), SME, TLA-1, VEB | Class A beta-lactamase |
| ccrA, IMP-1 group, IMP-12 group, IMP-2 group, IMP-5 group, NDM, VIM-1 group, VIM-13, VIM-7 | Class B beta-lactamase |
| ACC-1 group, ACC-3, ACT 5/7 group, ACT-1 group, CFE-1, CMY-10 Group, DHA, FOX, LAT, MIR, MOX | Class C beta-lactamase |
| OXA-10 Group, OXA-18, OXA-2 Group, OXA-23 Group, OXA-24 Group, OXA-45, OXA-48 Group, OXA-50 Group, OXA-51 Group, OXA-54, OXA-55, OXA-58 Group, OXA-60 | Class D beta-lactamase |
| ereB | Erythromycin resistance |
| QepA, QnrA, QnrB-1 group, QnrB-31 group, QnrB-4 group, QnrB-5 group, QnrB-8 group, QnrC, QnrD, QnrS | Fluoroquinolone resistance |
| ermA, ermB, ermC, mefA, msrA | Macrolide Lincosamide Streptogramin_b |
| Oprj, oprm | Multidrug resistance efflux pump |
| tetA, tetB | Tetracycline efflux pump |
| vanB, vanC | Vancomycin resistance |
| mecA | Beta-lactam resistance |
| lukF | Panton-Valentine leukocidin chain F precursor |
| spa | Immunoglobulin G binding protein A precursor |
