# Supplementary material for: Feed supplementation with potentiated zinc and/or tannin-rich extracts reduces ETEC infection severity and antimicrobial resistance genes in pig
Source: Front Vet Sci. 2025 Feb 21;12:1494103. doi: 10.3389/fvets.2025.1494103 (PMC11887510; doi:10.3389/fvets.2025.1494103)
Supplement: Supplementary file 3 [file Image_1.pdf]

Supplementary Material

Feed supplementation with potentiated zinc and/or tannin-rich extracts impact ETEC infection severity and antimicrobial resistance genes in pigs.

Catherine Ollagnier*, Johana Bellon, Maria-Rita Mellino, Nicolas Pradervand, Marco Tretola, Sebastien Dubois, Agathe Romeo, Olivier Desrues, Giuseppe Bee

*** Correspondence:** Corresponding Author: catherine.ollagnier@agroscope.admin.ch

S.3 VAS score in study 1

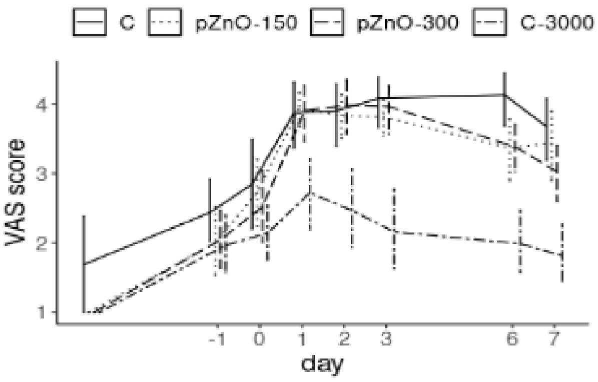

VAS

(40 mm long line)

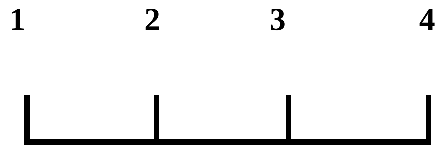

Figure S1: Fecal score and visual analogic score (VAS) in relation to experimental day in both studies. The fecal score was evaluated on a scale of 1 to 4. Normal molted feces were given a score of 1, while watery diarrhea was given a score of 4. The VAS consisted of a 40-mm-long horizontal line with numeric descriptors (1 to 4) every 10 mm to represent the fecal score. An observer marked the point on the line that best reflects the observed severity of the diarrhea. C-150 and C-3000: starter diets supplemented with 150 or 3000 mg/kg ZnO, respectively; pZnO-150 and pZnO-300: starter diets supplemented with 150 or 300 mg/kg of a potentiated ZnO source (HiZox®, Animine, Annecy, France), respectively.
